# White-Rot Fungal Pretreatment for High-Performance Bamboo-Derived Carbon-Based Supercapacitor Electrodes

**DOI:** 10.3390/molecules30163430

**Published:** 2025-08-20

**Authors:** Jian Zhang, Lin Lin, Tianyao Jiang, Jiaming Cao, Jun Zhang, Jing Qin, Hengnan Liang

**Affiliations:** 1 Key Laboratory of Wooden Materials Science and Engineering of Jilin Province, Beihua University, Jilin 132013, China; zhangjian_beihua@126.com (J.Z.); caojiaming_beihua@126.com (J.C.); zhangjun_beihua@126.com (J.Z.); qinjing_beihua@126.com (J.Q.); 2 College of Agricultural Engineering and Food Science, Shandong Research Center of Engineering and Technology for Clean Energy, Shandong University of Technology, Zibo 255000, China; jiangtianyao_sdut@126.com; 3 College of Physics, Jilin University, Changchun 130012, China; lhn@jlu.edu.cn

**Keywords:** fungal pretreatment, bamboo derived carbon, supercapacitor electrodes, white-rot fungi

## Abstract

Bamboo, as a rapidly renewable biomass material, has garnered significant attention in contemporary research due to its cost effectiveness as a viable source for supercapacitor electrode materials. However, untreated bamboo as an electrode material often leads to poor connectivity and uneven pore distribution. This study introduces a novel approach by using bamboo-derived biological carbon as a conductive substrate, subjecting it to carbonization through white-rot fungal pretreatment to enhance the pore structure and then loading it with nano-MnO_2_ sheets via a hydrothermal process. The result is a binderless, self-supporting supercapacitor electrode material, denoted as MnO_2_/hyphae/bamboo-derived carbon (HBC-2M). When compared to untreated bamboo carbon (HBC-0), HBC-2M exhibits an increased number of energy storage sites, enhanced electrolyte ion transport channels, and superior electrochemical performance. HBC-2M achieves a maximum mass-specific capacitance of 133.69 F·g^−1^ and a maximum area-specific capacitance of 2367.95 mF·cm^−2^ and retains approximately 87.46% of its capacitance after 2000 cycles. This research suggests a promising future for bamboo charcoal in supercapacitors.

## 1. Introduction

Electrochemical energy storage has become a pivotal technology in sustainable energy systems, with supercapacitors emerging as a particularly promising solution due to their unique combination of high power density, exceptional cycle stability, and rapid charge–discharge capabilities [1,2]. As a bridge between conventional capacitors and batteries, supercapacitors have attracted extensive research attention for applications requiring both energy and power density [3].

The performance of supercapacitors is fundamentally governed by their electrode materials. Among various candidates, biomass-derived carbon materials have gained prominence owing to their sustainability, low cost, and tunable porous structures [4,5]. However, conventional electrode fabrication often relies on metal current collectors and polymer binders, which introduce inactive mass and impede ion transport, ultimately compromising the device’s energy density [6]. This has spurred growing interest in binder-free, self-supporting electrodes that eliminate these parasitic components while maintaining mechanical integrity [7].

Sustainable low-cost natural resources, on the other hand, are conducive to the mass production of electrode materials [8,9]. Bamboo’s unique fiber structure is used as a raw material to produce carbon fiber; bamboo has a rapid growth rate, a brief maturity period, an abundance of raw materials, and a high yield [10]. Moreover, under nitrogen’s protection, bamboo can be carbonized at high temperatures to produce economical carbon materials with interconnected, multi-channel, porous structures [11]. White-rot fungal biopretreatment has emerged as a research focus in bamboo modification due to its environmental benignity and processing specificity [12]. This pretreatment employs specialized enzyme systems (including lignin peroxidase, manganese peroxidase, and laccase) to selectively degrade lignin components within bamboo, thereby forming a multilevel pore hierarchy within the cell walls. The process initiates with hyphal penetration through pits and intercellular spaces, triggering the preferential degradation of lignin in the middle lamella. This degradation subsequently expands into narrow-band regions of the secondary walls, ultimately generating micrometer-scale fungal channels and nanometer-scale inter-cellulose pores. This mechanism enables the precise modulation of the pore architecture. Such biological processing circumvents the use of strong acids/alkalis, aligning with green chemistry principles and opening new avenues for high-value utilization of bamboo [13]. The resulting multiscale pore network significantly enhances bamboo’s permeability, adsorption capacity, and interfacial bonding performance, demonstrating considerable potential for applications in high-performance composites and electrochemical energy storage devices. The use of white-rot fungi as a pretreatment to regulate bamboo-derived carbon materials for supercapacitors has not yet been documented [14].

This study combines the unique pore structure of bamboo carbon material with the advantages of ultra-high-capacity MnO_2_. Using bamboo as a conductive substrate, a self-supporting carbon fiber substrate was prepared by white-rot fungi etching and pyrolysis. MnO_2_ was then deposited directly on the prepared carbon fiber sheet using a simple hydrothermal method. The synthesized supercapacitor nanostructured hybrid electrode has an area-specific capacity of 2367.95 mF·cm^−2^ (current density is 1 mA·cm^−2^), a corresponding mass-specific capacitance of 138.27 F·g^−1^, and good cycling performance. This work may also provide an effective pretreatment method to avoid the environmental impact and huge waste of resources caused by the use of physicochemical activation methods and to develop possible high-performance supercapacitor electrodes.

## 2. Results and Discussion

### 2.1. SEM of Original Bamboo and Bamboo Pretreated with White-Rot Fungi

**Figure 1:**
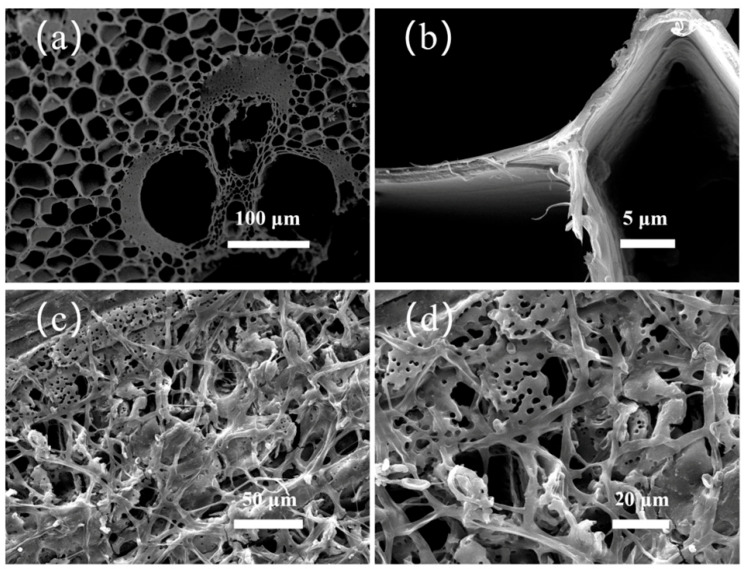
Top-view SEM images of (**a**,**b**) original bamboo and (**c**,**d**) bamboo pretreated with white-rot fungi for 2 weeks.

In comparison to other biomass materials, bamboo contains a higher concentration of nutrients, including starch and sugar, which renders it more susceptible to microbial degradation and leads to changes in its pore structure [15]. The native bamboo exhibits a well-ordered porosity, characterized by alternating brick-like (thick-walled) and fish-scale (thin-walled) microstructures in its cross-sectional SEM images (Figure 1a,b) [16]. Figure 1c,d shows bamboo pretreated with white-rot fungi for 2 weeks. After fungal treatment, the hyphae of white-rot fungi thrive on the inner walls of bamboo, creating an interconnected skeletal structure that augments the number of pathways for electron transport. Concurrently, the macropores were filled with hyphae growth, forming more small-sized pores than the original bamboo, thereby increasing the material’s specific surface area and enhancing its capacitive performance [17].

### 2.2. FTIR

**Figure 2:**
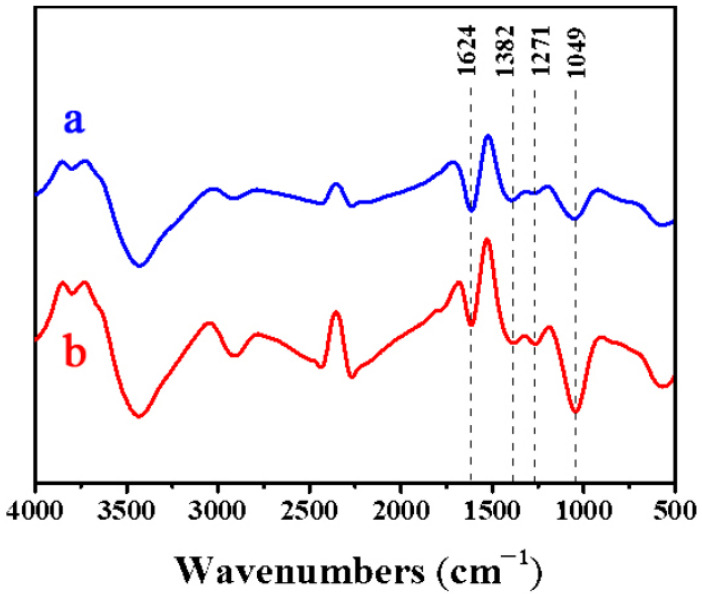
FTIR spectra of (**a**) original bamboo and (**b**) bamboo pretreated with white-rot fungi for 2 weeks.

Figure 2 shows the FTIR spectra of bamboo before and after treatment with white-rot fungi, and Table 1 lists the detailed information about the FTIR spectra [18,19]. The results indicate that the intensity of the characteristic peaks of cellulose and hemicellulose at 1049 cm^−1^ weakened after fungal degradation, while the characteristic peaks of lignin at 1624 cm^−1^, 1382 cm^−1^, and 1271 cm^−1^ showed the most significant reduction. This implies that white-rot fungi possess the ability to degrade bamboo’s lignin, cellulose, and hemicellulose to varying extents, with a stronger propensity for lignin degradation [20]. Changes in the absorption peaks indicate a dynamic process characterized by the gradual decomposition of carbohydrates (cellulose and hemicellulose) and lignin into shorter-chain hydrocarbons and linear compounds during the degradation process. The cyclic structures in lignocellulose are gradually broken down into linear compounds. However, as the fungus grows and utilizes the small-molecule products obtained from the degradation of lignocellulose and enzymes further degrade the small molecules, the content of short-chain hydrocarbons and linear compounds gradually decreases.

### 2.3. SEM of MnO_2_-, Hyphae-, and Bamboo-Derived Carbon

**Figure 3:**
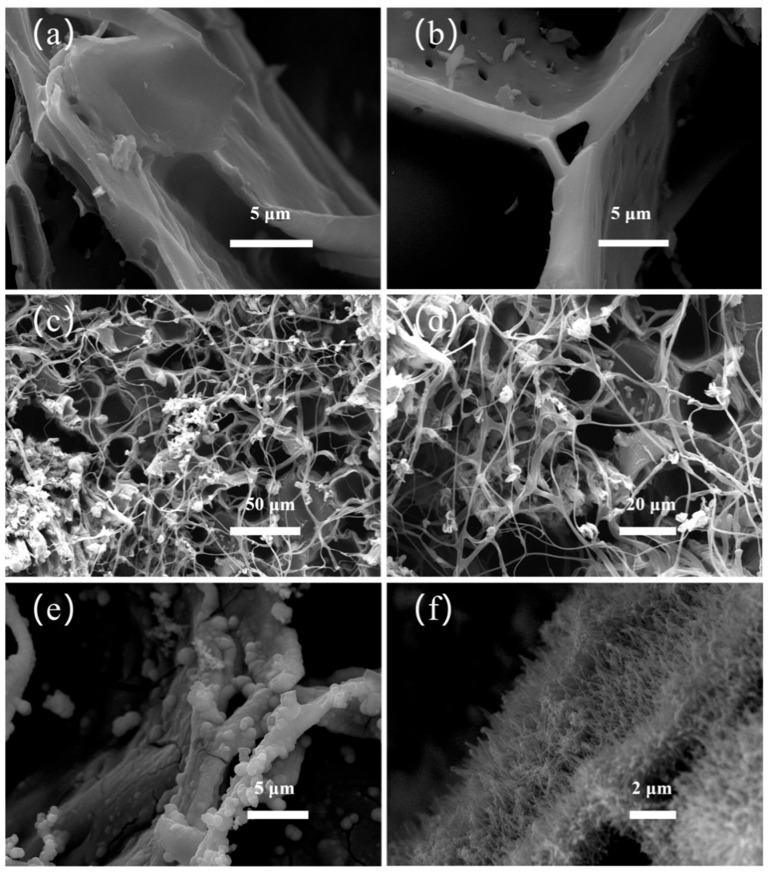
Top-view SEM images of (**a**,**b**) original bamboo-derived carbon (HBC-0), (**c**,**d**) hyphae/bamboo-derived carbon (HBC-2), and (**e**,**f**) MnO_2_/hyphae/bamboo-derived carbon (HBC-2M).

After carbonization, bamboo retains its original thin-walled structure and vascular bundle composition as a conductive substrate. SEM images (Figure 3a,b) show that the carbonized bamboo (HBC-0) still has smooth cell walls and intact pits. In the case of the hyphae/bamboo-derived carbon (HBC-2), the hyphae filled the cell cavity and formed numerous small-sized pores on the cell walls (Figure 3c,d). Additionally, the hyphae retained their original skeleton structure after carbonization, despite some volume reduction. The surface of MnO_2_/hyphae/bamboo-derived carbon (HBC-2M) contains a layer of uniform needle-like MnO_2_ nanoparticles (Figure 3e,f), which can increase the specific surface area and enhance the adsorption sites of electrolyte ions [21]. Moreover, MnO_2_ can improve the conductivity, a crucial guarantee for achieving the excellent capacitance performance of carbon materials [22]. This improved conductivity not only accelerates the migration rate of electrolyte ions within the pore channels but also reduces the equivalent series resistance of the capacitor, ultimately resulting in superior overall performance.

### 2.4. Pore Structure

**Figure 4:**
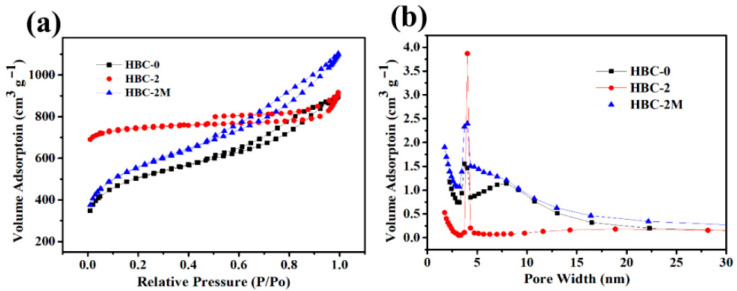
(**a**) Nitrogen adsorption–desorption isotherms and (**b**) pore size distributions of HBC-0, HBC-2, and HBC-2M.

The pore structure of bamboo-based porous carbon is closely related to the electrochemical performance of supercapacitors [23]. The effect of white-rot fungi pretreatment on the pore structure of bamboo-based porous carbon materials was investigated using the BET test. The nitrogen adsorption–desorption curves of bamboo-derived carbon electrodes prepared under different conditions are shown in Figure 4a. The adsorption capacity of fungus-pretreated bamboo charcoal is significantly higher than that of untreated bamboo charcoal, demonstrating that the activation process of white-rot fungi can play a crucial role in opening and creating pores and facilitating the introduction of a large number of mesopores.

Figure 4b displays the pore size distribution curves of bamboo charcoal samples prepared using three activation methods. The results suggested that all bamboo-based porous carbon materials (HBC-0, HBC-2, and HBC-2M) had a pore size centered at approximately 4 nm and exhibited a mesoporous structure. Compared with untreated bamboo charcoal materials, HBC-2, and HBC-2M have more mesopores due to the etching effect of white-rot fungi on the materials during the pretreatment process, resulting in a dual effect of pore formation and expansion. The specific surface area of the HBC-2 electrode material calculated from the nitrogen isothermal adsorption line is as high as 2369 m^2^·g^−1^, which is nearly 1.4 times that of the HBC-0 electrode material (1701 m^2^·g^−1^), indicating that the fungal pretreatment method can effectively increase the specific surface area of the material. The electrode material, loaded with manganese dioxide, has a high specific surface area of 1900 m^2^·g^−1^, suggesting that an increase in the composite material’s specific surface area can enhance the capacitor’s electrochemical performance [24].

### 2.5. XRD and Raman

**Figure 5:**
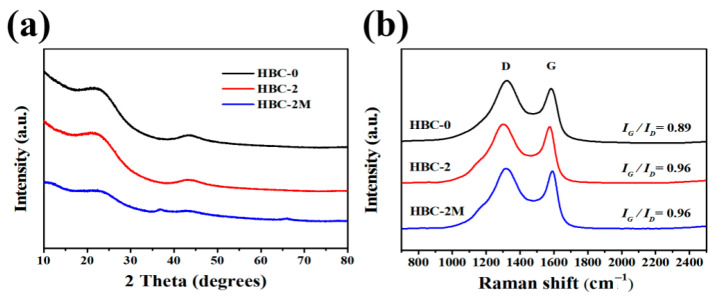
(**a**) XRD patterns of the HBC-0, HBC-2, and HWC-2M composites; (**b**) Raman spectra of the HBC-0, HBC-2, and HWC-2M composites.

The crystal structures of HBC-0, HBC-2, and HBC-2M were analyzed by X-ray diffraction (XRD) (Figure 5a). All three samples displayed two diffraction peaks at approximately 22.4° and 43.7°, corresponding to the (002) and (101) planes of carbon, indicating the presence of graphite-like structures. The peaks were broad and diffuse, indicative of low crystallinity and an amorphous nature of the materials. Comparing the XRD patterns of HBC-2M with those of HBC-0 and HBC-2, it can be seen that the peak positions of HBC-2M were closer to the theoretical graphite (002) peak, indicating a higher degree of graphitization. Additionally, two characteristic peaks observed at 36.8° and 66.2° in the XRD pattern of HBC-2M confirmed the successful formation of MnO_2_ [25], which matches the standard card for δ-MnO_2_ (JCPDS 80-1098) and is consistent with birnessite-type MnO_2_. Based on Bragg’s Law, the interlayer spacings for HBC-0, HBC-2, and HBC-2M are 3.967 Å, 4.015 Å, and 3.844 Å, respectively. In contrast, the theoretical interlayer spacing of graphite is 3.35 Å (2θ = 26.4°). The peak position of HBC-2M is closer to that of graphite, indicating a high degree of graphitization.

In the Raman spectra presented in Figure 5b, three different materials were observed to exhibit peaks in the vicinity of 1322 cm^−1^ and 1583 cm^−1^. The former peak, denoted as the D band, arises from low-symmetry carbon atoms at lattice defects or edges, whereas the latter peak, denoted as the G band, is generated by sp2 hybridized carbon atoms. The G band to D band intensity ratio (I_G_/I_D_) was employed as a measure of the graphitization degree and structural defects in the carbon materials. A higher I_G_/I_D_ ratio is indicative of a greater degree of graphitization. Notably, the I_G_/I_D_ values for HBC-2 and HBC-2M were found to increase to 0.96 from the initial value of 0.89 for HBC-0, suggesting that the graphitization degree of HBC-2 and HBC-2M was improved after pretreatment with white-rot fungi [26].

### 2.6. Electrochemical Performance

**Figure 6:**
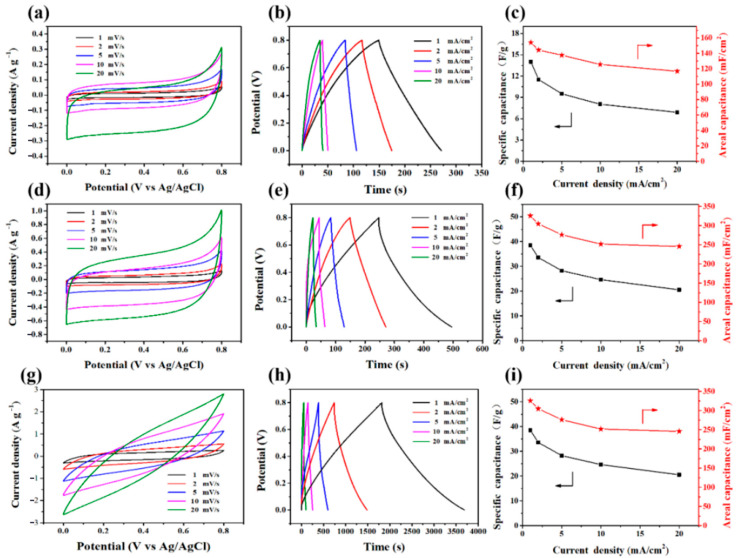
Electrochemical performance of HBC-0: (**a**) CV curves at various scan rates, (**b**) GCD curves at different current densities, and (**c**) rate performances. Electrochemical performances of HBC-2: (**d**) CV curves at various scan rates, (**e**) GCD curves at different current densities, and (**f**) rate performances. Electrochemical performance of HBC-2M: (**g**) CV curves at various scan rates, (**h**) GCD curves at different current densities, and (**i**) rate performances.

To further investigate the electrochemical performance of the electrode materials, cyclic voltammetry (CV) and galvanostatic charge–discharge measurements (GCD) were performed in a 1M Na_2_SO_4_ electrolyte. Figure 6a,d depicts the CV curves of the HBC-0 and HBC-2 electrodes at various scan rates (1, 2, 5, 10, and 20 mV·s^−1^), exhibiting ideal rectangular characteristics typical of electric double-layer capacitors. The more significant current density and integrated area of the CV curve for the HBC-2 electrode suggest that pretreatment with white-rot fungi can enhance the charge-storage capacity of the electrode materials. Figure 6b,e shows that the GCD curves of HBC-0 and HBC-2 have triangular and symmetrical shapes, indicating their excellent electrochemical reversibility. The capacitance of HBC-0 and HBC-2 nanocomposite electrodes was also estimated through GCD. The discharge area and specific capacitance are shown in Figure 6c,f. For the HBC-0 electrode, an area-specific capacitance of 154 mF·cm^−2^ was achieved at 1 mA·cm^−2^, and when the current density increased to 20 mA·cm^−2^, the capacity decreased to 116.75 mF·cm^−2^. The mass-specific capacitance calculated based on the total mass of the HBC-0 composite material decreased from a maximum value of 14 F·g^−1^ to 6.89 F·g^−1^. However, the HBC-2 electrode material achieved an area-specific capacitance of 325.41 mF·cm^−2^ at 1 mA·cm^−2^, corresponding to a maximum mass-specific capacitance of 133.69 F·g^−1^. The improvement in electrochemical performance of the HBC-2 electrode material was attributed to the fungal pretreatment, which improved the uneven distribution of pore size and poor connectivity issues [27].

The reaction attributed to the pseudo-capacitance is observed in the HBC-2M electrode material loaded with manganese dioxide (MnO_2_ + Na + e^−^ = MnOONa). Figure 6g indicates that the CV curve of HBC-2M strays from the rectangular shape, likely due to the polarization of the hydration process of sodium ions and the relatively low conductivity of MnO_2_ in the mixed electrode. Figure 6h illustrates the charge–discharge characteristics of the HBC-2M electrode between 0 and 0.8 V. During charging and discharging processes at various current densities (1–20 mA·cm^−2^), the HBC-2M electrode exhibits nearly symmetrical charge and corresponding discharge curves, indicating excellent capacitive behavior and highly reversible Faraday reactions between Na and MnO_2_ [28]. Notably, the discharge time of HBC-2M is longer than that of HBC-0 and HBC-2 electrodes. This behavior is consistent with specific capacitance, as the discharge time is proportional to the electrode’s specific capacitance. The specific capacitance of the HBC-2M electrode was calculated through Figure 6i. At current densities of 1, 2, 5, and 10 mA·cm^−2^, the HBC-2M electrode exhibited area capacities of 2367.95, 1884.67, 1471.1, and 1355.7 mF·cm^−2^, respectively, which correspond to mass-specific capacitances of 133.69, 120.97, 80.14, and 56.28 F·g^−1^, respectively. At a current density of 20 mF·cm^−2^, the material maintained an area-specific capacitance of 1283.4 mF·cm^−2^. Compared to the HBC-0 and HBC-2 electrodes, the HBC-2M electrode possesses a distinct three-dimensional needle-like structure. The pore channels, broken by white-rot fungi, facilitate better loading of MnO_2_, resulting in a larger specific surface area and enhanced contact between the electrode and electrolyte. The enhancement in capacitance is attributed to the effective control of pore structure and the pseudo-capacitive redox reaction of MnO_2_.

**Figure 7:**
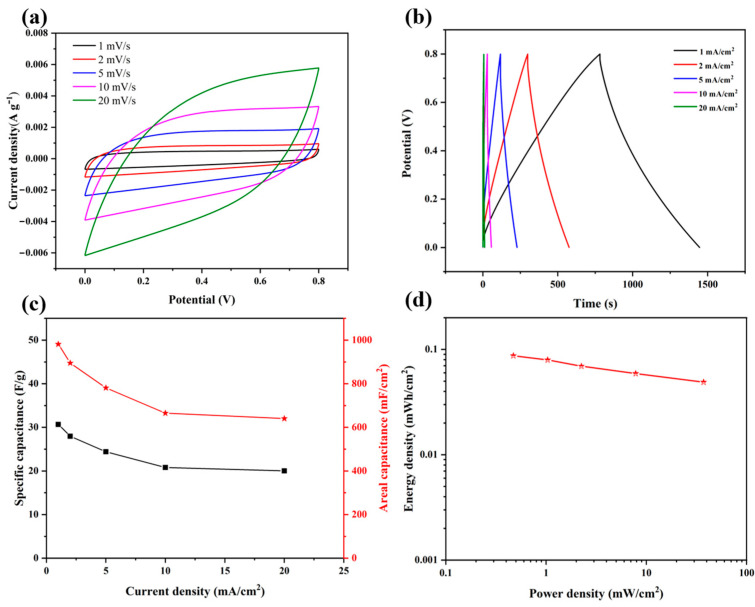
Electrochemical performance of the HBC-2M assembled supercapacitor. (**a**) CV curves at different scanning rates, (**b**) GCD curves at different current densities, (**c**) rate performance, and (**d**) energy density versus power density curve.

To explore the electrochemical performance of HBC-2M at the device level and to investigate its significance at the practical energy storage level, two identical HBC-2M electrodes were taken as working electrodes, and a 1M Na_2_SO_e_ solution was used as the electrolyte, with a cellulose membrane as the separator to assemble a symmetrical supercapacitor. Electrochemical measurements were conducted under a dual-electrode system. Figure 7 shows the electrochemical performance of symmetrical supercapacitors. The CV curves in Figure 7a show that at different scanning rates, all CV curves are quasi-rectangular without obvious deviation. The polarization of the hydrated sodium ion desolvation process in the three-electrode system and the low conductivity phenomenon caused by MnO_2_ in the mixed electrode are stabilized under the two-electrode system. According to the GCD curves in Figure 7b, it can be seen that at different current densities, all GCD curves show intentional symmetry, indicating that it has good capacitive performance and excellent reversibility. The area-specific capacitances of the supercapacitors assembled with HBC-2M at current densities of 1, 2, 5, 10, and 20 mA cm^−2^ are 981.5, 895.12, 781.25, 665.22, and 640.3 mF cm^−2^, respectively, while the mass-specific capacitances are 30.6, 27.97, 24.41, 20.73, and 18.09 F/G. Even at a current density of 20 mA cm^−2^, the area-to-capacitance ratio is still 65.23% of that at 1 mA cm^−2^, demonstrating excellent rate performance. Figure 7d shows the energy density and power density curves of supercapacitors. HBC-2M has an energy density of 0.087 mWh cm^−2^ at a power density of 0.47 mW cm^−2^ and still has an energy density of 0.048 mWh cm^−2^ even at a power density of 37.25 mW cm^−2^. This indicates that HBC-2M still has good electrochemical performance at the device level and practical application value at the device level.

## 3. Materials and Methods

### 3.1. Materials

The sawdust from bamboo and pinus massoniana sapwood was purchased by Wenxi Machinery Co., Ltd. (Lianyungang, China). White-rot fungi were purchased from Beina Chuanglian Biotechnology Co., Ltd. (Suzhou, China). Potassium permanganate, glucose, and agar were all acquired from Kaimio Chemical Reagent Co., Ltd. (Tianjin, China), Damao Chemical Reagent Factory (Tianjin, China), and Zhiyuan Chemical Reagent Co., Ltd. (Tianjin, China). Sodium sulfate was purchased from Quanrui Reagent Co., Ltd. (Jinzhou, China). All the chemicals mentioned above were used without further purification. Brown sugar, corn flour, sand, and potatoes are purchased at the local farmers’ market.

### 3.2. Methods

#### 3.2.1. Preparation of River Sand Sawdust Media

For the inoculation media preparation, 200 g of fresh potatoes was weighed; peeled, washed, and cut into pieces; and then placed in 1000 mL of boiling water for 30 min. This mixture was filtered with gauze; then, 20 g of glucose was added, and water was added to 1000 mL. After fully dissolving, it was ready for use as a maltose solution.

To a wide-mouthed triangular flask, 150 g of dry river sand, 15 g of sawdust of Masson pine sapwood, 8.5 g of corn flour, and 1 g of brown sugar were added. After mixing well and leveling, three pieces of fodder wood were placed into the culture medium, to which 100 mL of maltose solution was added. The flask was plugged with absorbent cotton and wrapped in kraft paper before being placed into a steam sterilizer for sterilization at 121 °C for 1 h. The river sand sawdust culture medium was then obtained, and upon cooling, it was used for inoculating white-rot fungi.

#### 3.2.2. Test Bacteria Inoculation and Cultivation

An inoculating loop was used to cut a 5 mm diameter hyphal piece (enriched with agar medium) from the hypha growing in the Petri dish, and it was inserted into the middle section of the river sand sawdust culture medium (approximately 5 mm deep from the surface layer). After the inoculated culture flasks were sealed, they were placed in a constant temperature and humidity chamber at a temperature of (28 ± 2) °C and a relative humidity of 75% to 85% until the surface of the culture medium in the flask was covered with hypha. Then, the sample was infected with bacteria. All procedures were conducted under sterile conditions to prevent contamination by adulterating bacteria.

#### 3.2.3. Inoculation of Samples

The bamboo was placed into an oven at a temperature of 103 ± 2 °C until it reached a constant weight and then cooled down for later use. Then, it was placed in an autoclave and maintained at a normal pressure for 30 min, allowing the sample’s moisture content to reach 40% to 60%. The cooled samples were placed on hypha-covered feeding wood in sterile conditions, with each bottle containing three samples (with the direction of the texture perpendicular to the direction of hyphal growth). The culture flask was placed in a constant temperature and humidity chamber under the aforementioned conditions for a decomposition time of 2 weeks. The entire inoculation process was conducted on a clean bench with meticulous disinfection procedures during the operation to prevent the introduction of foreign bacteria.

#### 3.2.4. Preparation of Hypha/Bamboo-Based Carbon Material

After 2 weeks, the samples were taken out, and the surface hypha and impurities were gently scraped off. They were then dried to a constant weight in a drying oven at 103 ± 2 °C. Subsequently, the bamboo sample eroded by white-rot fungi was placed in a tube furnace under nitrogen protection. From there, it was heated at a rate of 5 °C·min^−1^ to 500 °C for 1.5 h and then to 800 °C for 2 h for complete pyrolysis. Finally, it was cooled from 5 °C·min^−1^ to 500 °C and finally to room temperature naturally, yielding a hypha/bamboo-based carbon material.

#### 3.2.5. Preparation of MnO_2_/Hypha/Bamboo-Based Carbon Material

**Figure 8:**
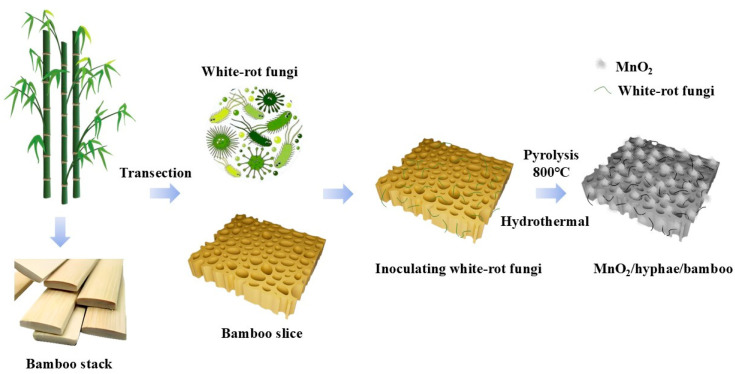
Schematic diagram of the full synthetic characterization process.

A mixed solution of 50 mL of KMnO_4_ (0.1 M) and Na_2_SO_4_ (0.1 M) was stirred at room temperature until complete dissolution. The hyphae-loaded charcoal chips were soaked in the mixed solution, and the solution containing the above mixture was transferred to a 100 mL high-pressure reactor and stored in a 150 °C oven for 1.5 h. Then, the mixture was repeatedly washed with deionized water and dried in a 60 °C vacuum-drying oven for 3 h to obtain the MnO_2_/hypha/bamboo-based carbon composite electrode material. The areal mass loadings of HBC-0, HBC-2, and HBC-2M were 11.0 ± 0.5 mg·cm^−2^, 10.2 ± 0.4 mg·cm^−2^, and 13.7 ± 0.6 mg·cm^−2^, respectively. The schematic diagram of the full synthetic characterization process is shown in Figure 8.

#### 3.2.6. Preparation of Comparative Samples

This is a comparative experimental study on the influence of white-rot fungus hypha etching on bamboo in composite electrode materials. The unmodified raw carbonized bamboo sample obtained from the experiment is denoted as HBC-0, the sample obtained after 2 weeks of fungal etching and carbonization is denoted as HBC-2, and the sample obtained after 2 weeks of fungal etching and carbonization of hypha/porous bamboo charcoal/MnO_2_ composite material loaded with manganese dioxide is denoted as HBC-2M.

#### 3.2.7. Characterization

The functional group variations in the samples were analyzed using a Tensor 27 Fourier Transform Infrared spectrometer (FT-IR, Bruker, Ettlingen, Baden-Württemberg, Germany) over a wavenumber range of 7800 to 350 cm^−1^ with a resolution of 0.5 cm^−1^. The morphology of the cross-sections was observed using an FEI Quanta 200 scanning electron microscope (SEM, FEI Quanta 200, Fremont, CA, USA). Parameters including pore size distribution and porosity were tested using an AutoPore IV 9500 mercury intrusion porosimeter and an ASAP 2020 surface area and porosity analyzer (BET, Micromeritics, Norcross, GA, USA). X-ray diffraction analysis of the samples’ structural properties was performed on a X-ray diffractometer using Cu Kα radiation (λ = 0.1542 nm) as the source (XRD, Rigaku DX2500, Akishima, Tokyo, Japan). Further quantitative analysis of the material’s degree of graphitization was conducted using a Renishaw InVia Raman spectrometer (Raman, Renishaw, Wotton-under-edge, Gloucestershire, UK).

#### 3.2.8. Electrochemical Testing

Electrochemical performance tests, including cyclic voltammetry (CV), galvanostatic charge–discharge (GCD), and electrochemical impedance spectroscopy (EIS), were conducted using a CHI760E electrochemical workstation (Chenhua Instruments Co., Ltd., Shanghai, China). Measurements employed a three-electrode system in a 1 M Na_2_SO_4_ electrolyte, with Pt foil as the counter electrode and an Ag/AgCl electrode as the reference electrode. The working electrodes consisted of free-standing electrode materials (approximately 10 × 10 × 0.1 mm) prepared as described previously. The electrochemical performance of HBC-0, HBC-2, and HBC-2M electrode materials was evaluated using this configuration. The CV and GCD tests utilized a voltage window of 0 to 0.8 V, while EIS measurements covered a frequency range of 0.01 to 10^5^ Hz. The specific capacitance (gravimetric and areal) was calculated using the following formula:

$$C_{m}=\frac{I\Delta t}{m\Delta V}$$

$$C_{s}=\frac{I\Delta t}{s\Delta V}$$

where *I* (A) is the discharge current, Δ*t* (s) is the discharge time, Δ*V* (V) is the potential window, *s* (cm^2^) is the electrode area exposed to the electrolyte, and *m* (g) is the mass of the active material on the electrode.

## 4. Conclusions

In this work, a new self-supporting porous bamboo carbon/MnO_2_ composite electrode material was developed. An eco-friendly activation process using fungi and a straightforward carbonization method were employed to create a bamboo-based biocarbon electrode material capable of supporting MnO_2_ adhesion. The material exhibited a high capacitance retention rate of 87.5% after 2000 cycles, demonstrating its significant potential for use in supercapacitors. This outstanding performance can be attributed to the favorable pore structure of the material, which facilitates ion transport. The innovative biological pretreatment method for regulating the pore structure of the electrode material provides valuable insights into the development of environmentally friendly and sustainable energy storage devices. This cost-effective and porous composite material can serve as a benign matrix material when combined with other electrochemically active substances, making it possible to design high-performance energy storage devices. This work demonstrates the promise of fungal-activated bamboo carbon/MnO_2_ composites for supercapacitors, but several strategic avenues warrant further exploration. Regarding the mechanistic optimization of bio-interfaces, probing molecular-level interactions between fungal-etched carbon surfaces and metal oxides should be explored to guide adhesion engineering. Regarding sustainable scalability, industrial-scale fungal cultivation parameters should be validated to reduce pretreatment time. In addition, cradle-to-gate lifecycle impacts versus conventional KOH activation should be assessed.

## Figures and Tables

**Table 1:** Detailed information of the FTIR spectra.

Wavenumber (cm^−1^)	Primary Assignment	Bond/Functional Group Vibration Type
1049	Cellulose /hemicellulose	C–O–C ether bond stretching vibration C–O stretching vibration (primary/secondary alcohols) [18]
1624	Lignin	Aromatic ring C=C stretching vibration (benzene ring skeleton) Conjugated carbonyl C=O stretching vibration
1382	Lignin	Phenolic O–H in-plane bending vibration [19] Aliphatic C–H bending vibration (–CH_3_, –CH_2_–)
1271	Lignin (Guaiacyl unit)	Guaiacyl ring C–O stretching vibration (aromatic ether Ar–O–CH_3_)

## Data Availability

Data will be made available upon request.
